# The Role of Serum Beta-Human Chorionic Gonadotropin (β-hCG) in Differentiating Benign and Malignant Breast Lesions at a Tertiary Care Center in Jharkhand

**DOI:** 10.7759/cureus.88294

**Published:** 2025-07-19

**Authors:** Neyaz Ahmad, Khushboo Rani, Zenith Kerketta, Krishna Murari, Anish Baxla, Ujala Murmu, Amit Nishant, Shreya

**Affiliations:** 1 General Surgery, Rajendra Institute of Medical Sciences, Ranchi, IND

**Keywords:** benign and malignant breast lesions, beta-human chorionic gonadotropin (β-hcg), metastatic breast disease, screening of breast cancer, tumor marker

## Abstract

Breast lumps are often the earliest clinical sign of breast cancer and a primary focus of early detection efforts. Tumor markers like serum beta-human chorionic gonadotropin (β-hCG) offer a potentially simple and cost-effective means for disease monitoring and management. However, the diagnostic value of such markers in breast cancer remains uncertain, particularly due to low sensitivity in early stages.

This study aimed to assess whether serum β-hCG levels can distinguish between benign and malignant breast lesions and evaluate their utility as a tumor marker in breast cancer patients. A total of 180 patients presenting with breast lumps were included through convenient sampling. Serum β-hCG levels were measured and correlated with histopathological findings. Among breast cancer patients, those with confirmed metastases were analyzed separately.

Of all participants, 54.44% had benign lesions and 45.56% had malignant tumors. Elevated β-hCG levels were found in 34.15% of malignant cases, compared to only 1.02% of benign cases, a statistically significant difference. The mean β-hCG level was significantly higher in malignant cases (3.81 ± 2.19 mIU/mL) than in benign ones (1.66 ± 1.10 mIU/mL). Furthermore, 87.5% of metastatic patients had elevated β-hCG levels, compared to only 5.13% among nonmetastatic patients, with a corresponding rise in mean levels.

These findings suggest that elevated serum β-hCG is associated with breast cancer, particularly in advanced stages and metastatic disease. While promising as a noninvasive biomarker, its limited sensitivity in early detection and the single-center, small-scale design of this study underscore the need for further research.

## Introduction

In 2022, an estimated 2.3 million new breast cancer cases and 666,000 breast cancer-related deaths occurred globally, accounting for 23.8 % and 15.4 % of all cancer cases and deaths in women, respectively [1]. The role of hormonal factors in breast cancer has been verified since the observation that metastatic breast cancer regressed after oophorectomy. Estrogens were the first sex hormones identified to induce breast cancer growth by binding to the nuclear estrogen receptor α [2]. Subsequent studies have also suggested a link between male sex hormone levels and breast cancer risk [3]. Prolactin, a reproductive peptide hormone, has been associated with breast cancer development in postmenopausal women [4].

The significance of tumor markers in breast cancer management has been a topic of debate owing to their low sensitivity in early-stage disease [5]. The American Society of Clinical Oncology (ASCO) has formulated guidelines for using tumor markers in the prevention, screening, treatment, and surveillance of breast cancer [6]. There are 13 categories of breast tumor markers. The tumor markers with certain clinical utility include CA 15-3, CA 27.29, carcinoembryonic antigen (CEA), estrogen receptor (ER), progesterone receptor (PR), human epidermal growth factor receptor 2 (HER2), urokinase plasminogen activator (uPA), plasminogen activator inhibitor 1 (PAI-1), and multiparameter gene expression assays [6]. However, markers such as P53, cathepsin D, cyclin E, and nestin lack sufficient evidence for their routine clinical use [5].

Human chorionic gonadotropin (hCG) is composed of two subunits (α and β). Syncytiotrophoblast cells of the placenta during pregnancy are the primary source for β-hCG production [7]. This hormone is responsible for the production of progesterone from the corpus luteum and hence is very essential for maintaining pregnancy. The association of hCG and breast cancer remains controversial. Ectopic expression of the hCG β subunit by breast tumors has been used as a biomarker for malignancy. On the other hand, hCG has also been formulated as a ligand media for delivering toxic drugs to target the luteinizing hormone (LH)/hCG receptor, which is expressed in malignant breast tissue. Some studies suggest that hCG may have a protective effect against breast cancer, prompting proposals for its use as a prophylactic measure in non-pregnant women [8].

Research has revealed that the β subunit of hCG can have an independent function, and β-hCG is also found in malignancies of the stomach, pancreas, cervix, liver, and breast [9]. Ectopic production of hCG occurs in various tumors, including breast cancer, and is linked to more aggressive tumor behavior [10]. There are also some reports suggesting the presence of serum β-hCG in phyllodes tumors [11]. Studies analysing peripheral blood from breast cancer patients were reported to have a varying range of elevated hCG levels [9]. Elevated serum levels of hCG and its metabolites are generally considered indicators of poor prognosis [12]. Timely identification of primary and metastatic breast cancer is crucial for early clinical decisions and initiating required treatment when the tumor load is minimal. This not only enhances the effectiveness of adjuvant therapy but also improves patient outcomes [13].

This study tries to find out the proportion of elevated β-hCG levels in different breast pathologies and assess its potential as a serum marker to distinguish between benign and malignant lesions. The following study also explores the possibility of using serum β-hCG for early detection of metastases in breast cancer patients. Since estimation of serum β-hCG is easily available, it seeks to explore its role as a tumor marker in breast cancer screening, diagnosis, and management.

## Materials and methods

This hospital-based analytical cross-sectional study was conducted in the General Surgery Department of Rajendra Institute of Medical Sciences (RIMS), Ranchi, India, over a period spanning from August 2023 to April 2024. A total of 180 female patients with breast lumps scheduled for surgical treatment were enrolled using a convenience sampling method. The study included women aged 18 years and above with a confirmed diagnosis of a breast lump requiring excision, while pregnant women and males with breast conditions were excluded. Chemiluminescent immunoassay (CLIA) was used for β-hCG assay.

Preoperative venous blood samples were collected from all participants, and serum β-hCG levels were obtained. The data were analysed using IBM SPSS Statistics for Windows, Version 25 (Released 2017; IBM Corp., Armonk, New York, United States) and MS Excel (Microsoft Corporation, Redmond, Washington, United States). Categorical variables were presented as frequency tables, and continuous variables were expressed as mean ± standard deviation or median (minimum, maximum). Normality was assessed using the Shapiro-Wilk test, and Quantile-Quantile (QQ) plots guided the choice between parametric and nonparametric tests. Fisher’s exact test was employed to examine associations between categorical variables, while the Mann-Whitney U test compared serum β-HCG levels across lesion types and metastatic status. Logistic regression and receiver operating characteristic (ROC) curves were used to evaluate the predictive ability of serum β-HCG for lesion type, with optimal cutoff values determined via the Youden index. A p-value ≤ 0.05 was considered statistically significant for all analyses. Approval for the study was obtained from the Institutional Ethics Committee of RIMS (approval no. 281, dated 16/08/2023).

## Results

The data consists of measurements from 180 subjects. The following table gives the distribution of subjects according to different variables (Table 1). The mean age of subjects is 39.19 ± 14.74 years (median: 40; range: 18-87). The mean age at menarche was 12.09 ± 1.62 years (median: 12; range: 8-16), while among 36 menopausal individuals, the mean age at menopause was 51.50 ± 2.61 years (median: 52; range: 43-56). A vast majority (98.33%) had no family history of the condition, and only 1.67% reported a positive family history. Regarding lifestyle factors, 6.11% of subjects reported alcohol or tobacco use. Unilateral involvement (90.56%) was more common than bilateral (9.44%), and multiple lesions were present in 7.78% of cases. Lymph node involvement was observed in 31.11% of subjects, while metastases were noted in 13.33%. Based on the Ultrasound Breast Imaging Reporting and Data System (USG BIRADS) classification, most lesions were categorized as BIRADS 2 (33.33%), followed by BIRADS 4 (28.89%), BIRADS 3 (25%), and BIRADS 5 (12.78%). Serum β-hCG levels were negative in 83.89% of cases, while 16.11% showed raised levels, with a mean value of 2.64 ± 1.99 (median: 2.10; range: 0.10-11.70). Among all cases, 54.44% were benign lesions, whereas 45.56% were malignant.

**Table 1:** Distribution of subjects according to different variables BIRADS: Breast Imaging and Data Reporting System; β-hCG: beta-human chorionic gonadotropin; SD: standard deviation

Variables	Subcategory	Number of subjects (%)
Age	Mean ± SD	39.19 ± 14.74
	Median (min, max)	40 (18, 87)
Age at menarche	Mean ± SD	12.09 ± 1.62
	Median (min, max)	12 (8, 16)
Age at menopause (n = 36)	Mean ± SD	51.50 ± 2.61
	Median (min, max)	52 (43, 56)
Family history	No	177 (98.33%)
	Yes	3 (1.67%)
Alcohol/tobacco	No	169 (93.89%)
	Yes	11 (6.11%)
U/L or B/L	B/L	17 (9.44%)
	U/L	163 (90.56%)
Multiple lesions	No	166 (92.22%)
	Yes	14 (7.78%)
Lymph node involvement	No	124 (68.89%)
	Yes	56 (31.11%)
Metastases	No	156 (86.67%)
	Yes	24 (13.33%)
Ultrasonography BIRADS	2	60 (33.33%)
	3	45 (25%)
	4	52 (28.89%)
	5	23 (12.78%)
Serum β-hCG	Negative	151 (83.89%)
	Raised	29 (16.11%)
	Mean ± SD	2.64 ± 1.99
	Median (min, max)	2.10 (0.10, 11.70)
Lesion type	Benign	98 (54.44%)
	Malignant	82 (45.56%)

**Figure 1:**
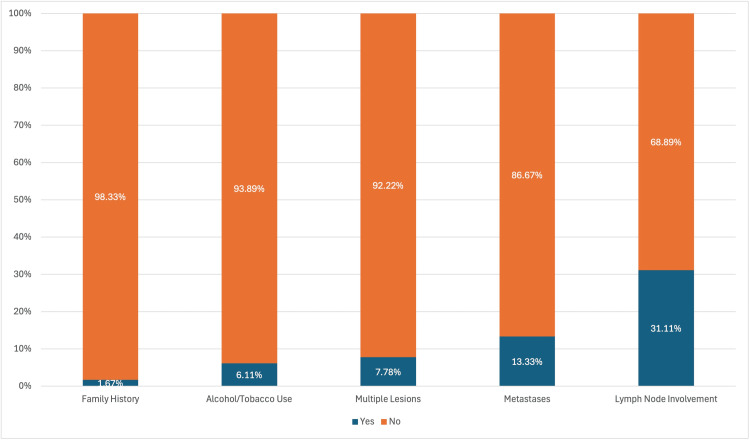
Comparison based on family history, alcohol/tobacco use, number of lesions, status of metastases, and lymph node involvement

In this group of 180 women with either benign or malignant breast lesions, the overwhelming majority (98.33%) had no known family history of breast disease, indicating that most cases were likely sporadic rather than inherited. Only a small percentage (6.11%) reported alcohol or tobacco use, suggesting these habits were not widespread contributors to their condition. A total of 92.22% of the cases were solitary, with multiple lesions being relatively rare. While many patients showed no signs of lymph node involvement, a significant portion were positive for lymph nodes (31.11%). Additionally, 13.33% of patients showed metastatic spread despite receiving standard diagnostic and treatment protocols, reflecting a subset with advanced disease. The majority of patients (163 individuals, 90.56%) had involvement of a single breast, indicating that unilateral presentation was far more common in this study population (Figure 1).

**Figure 2:**
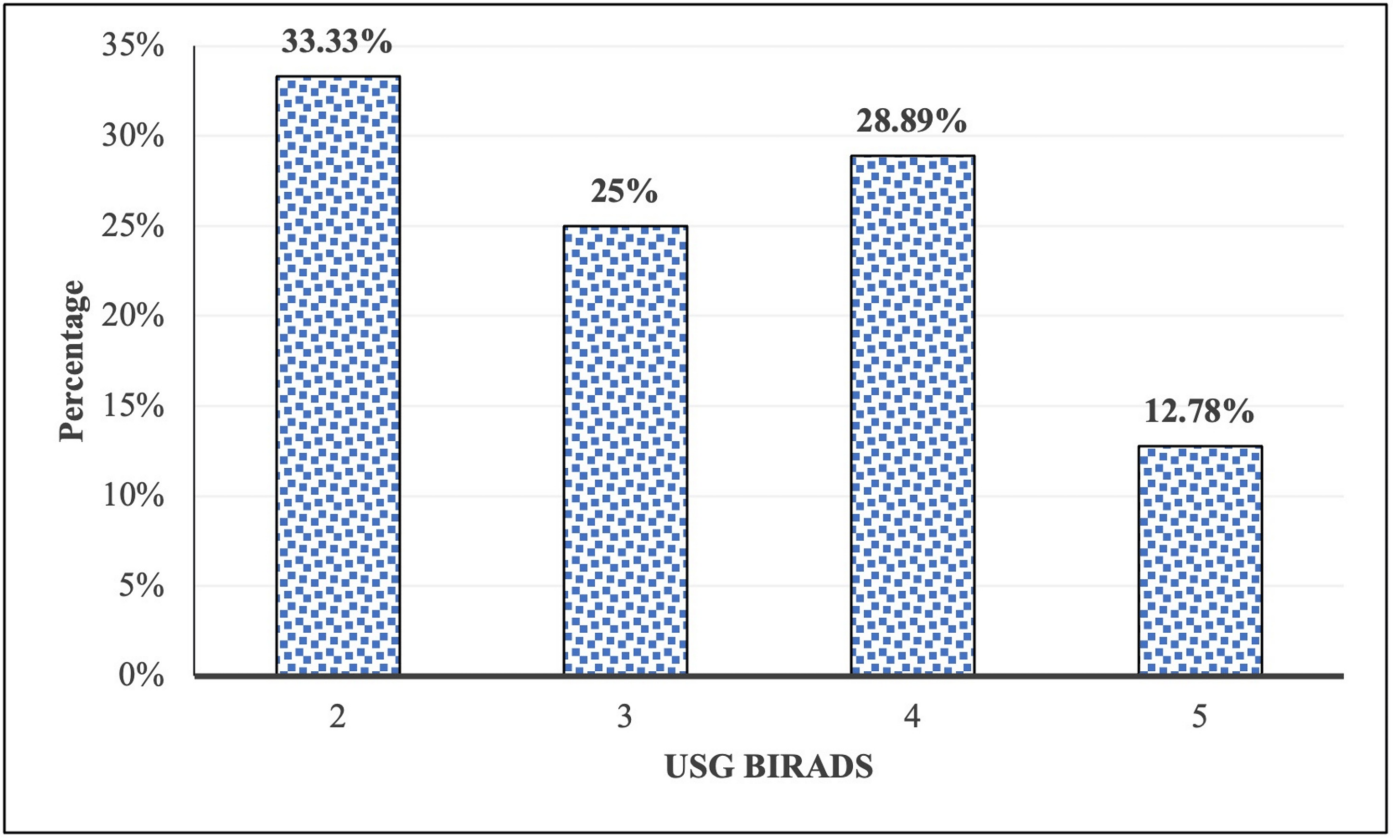
: Distribution of subjects according to USG BIRADS USG BIRADS: Ultrasound Breast Imaging Reporting and Data System

Out of the 180 cases of benign and malignant breast lesions, 60 patients (33.33%) were classified as BIRADS 2, representing benign findings; 45 patients (25%) were placed in BIRADS 3, indicating probably benign lesions; 52 patients (28.89%) fell under BIRADS 4, suggesting suspicious abnormalities; and 23 patients (12.78%) were categorized as BIRADS 5, reflecting a high likelihood of malignancy (Figure 2).

**Figure 3:**
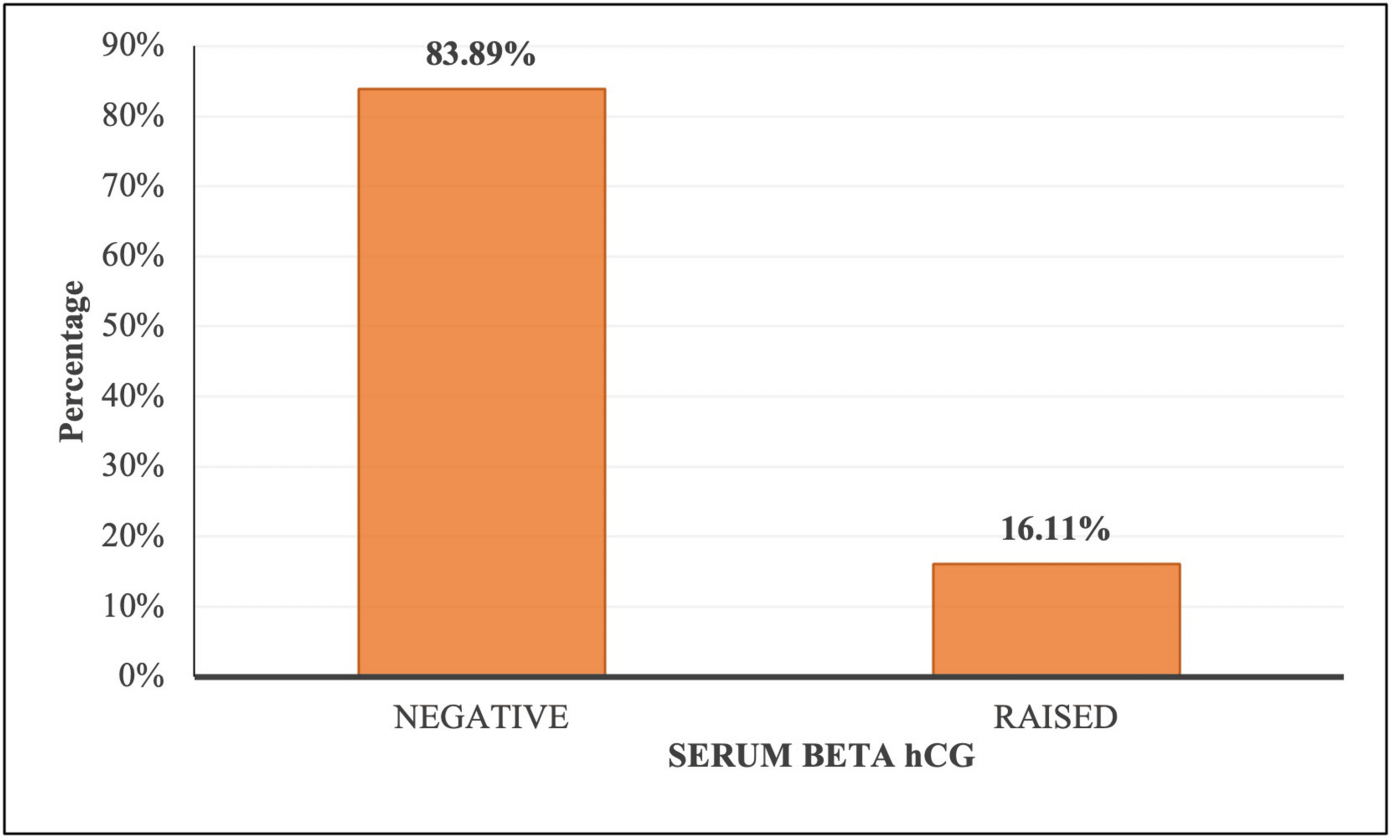
Distribution of subjects according to serum β-hCG β-hCG: beta-human chorionic gonadotropin

Out of 180 cases of benign and malignant breast lesions, β-hCG was raised in 29 (16.11%) patients, whereas it was negative in 151 (83.89%) patients (Figure 3).

**Figure 4:**
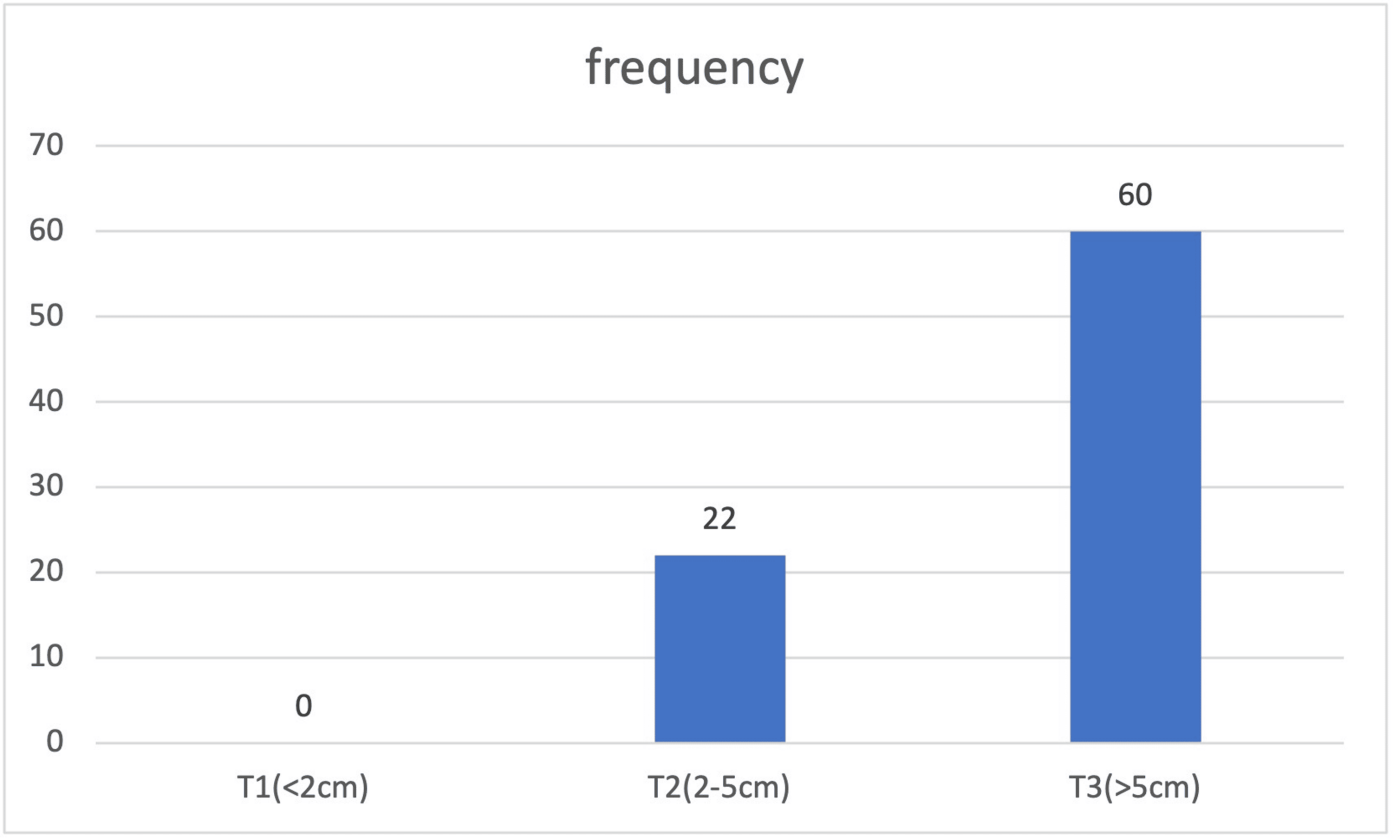
Distribution of malignant cases as per size

Among the 82 malignant breast cancer cases, none had tumors smaller than 2 cm, indicating that no early-stage small tumors were present. Twenty-two cases were classified as T2 stage, with tumor sizes between 2 cm and 5 cm, representing moderate tumor growth. The majority, 60 cases, were categorized as T3 stage, with tumors larger than 5 cm, suggesting more advanced disease and potentially aggressive tumor characteristics. This distribution points to a tendency for larger tumors at diagnosis in this group, underscoring the need for earlier detection and treatment (Figure 4).

**Figure 5:**
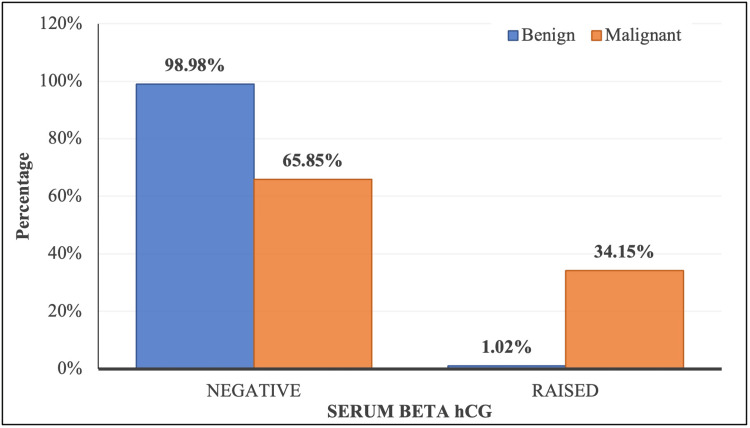
Distribution of serum β-hCG over lesion type β-hCG: beta-human chorionic gonadotropin

**Figure 6:**
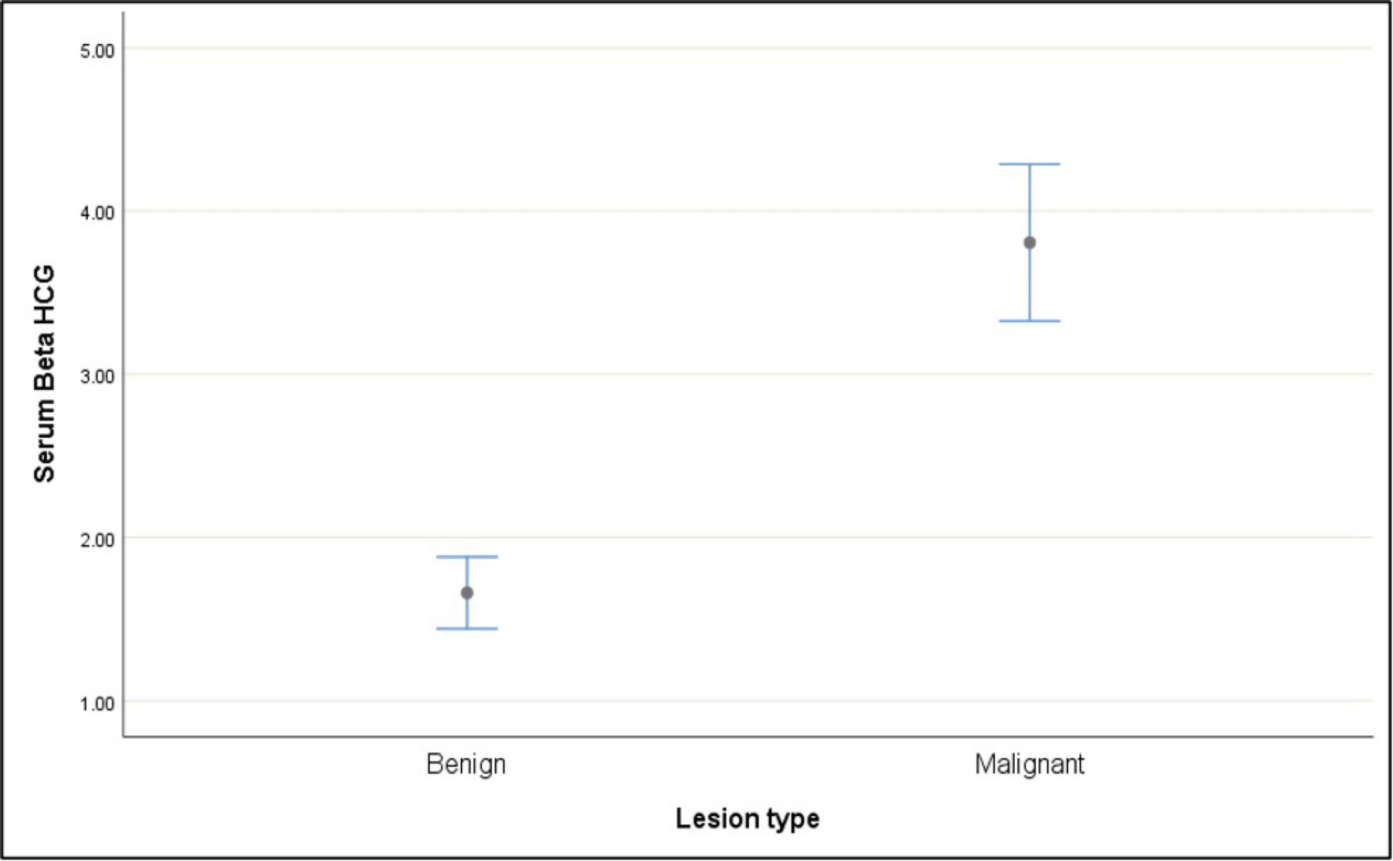
Mean plot of serum β-hCG over lesion type β-hCG: beta-human chorionic gonadotropin

Among subjects with benign lesions, 98.98% had negative β-hCG levels, while only 1.02% had raised levels. In contrast, 34.15% of malignant cases exhibited raised β-hCG levels, indicating a significantly higher proportion (Table 2). The mean serum β-hCG level was 1.66 ± 1.10 in benign cases (median: 1.40; range: 0.10-9.18) and significantly higher at 3.81 ± 2.19 in malignant cases (median: 3.45; range: 0.10-11.70) (Figures 5, 6). Both Fisher’s exact test and the Mann-Whitney U test showed a statistically significant difference in serum β-hCG levels between benign and malignant lesions (p-value < 0.001). These findings suggest a potential association between elevated β-hCG levels and malignancy.

**Table 2:** Comparison of serum β-hCG over lesion type β-hCG: beta-human chorionic gonadotropin; F: Fisher’s exact test; MW: Mann-Whitney U test; SD: standard deviation; * indicates statistical significance

Serum β-hCG	Lesion type		p-value
	Benign	Malignant	
Negative	97 (98.98%)	54 (65.85%)	<0.001^F^*
Raised	1 (1.02%)	28 (34.15%)	
Mean ± SD median (min, max)	1.66 ± 1.10 1.40 (0.10, 9.18)	3.81 ± 2.19 3.45 (0.10, 11.70)	<0.001^MW^*

Among those without metastases, 94.87% had negative β-hCG levels, while only 5.13% had raised levels. In contrast, a significantly higher proportion (87.5%) of subjects with metastases exhibited raised β-hCG levels. The mean serum β-hCG level was 2.09 ± 1.37 in subjects without metastases (median: 1.90; range: 0.10-9.18) and significantly higher at 6.19 ± 1.72 in those with metastases (median: 5.90; range: 3.20-11.70) (Table 3).

**Table 3:** Comparison of serum β-hCG over metastases β-hCG: beta-human chorionic gonadotropin; F: Fisher’s exact test; MW: Mann-Whitney U test; SD: standard deviation; * indicates statistical significance

Serum Β-hCG	Metastases		p-value
	No	Yes	
Negative	148 (94.87%)	3 (12.5%)	<0.001^F^*
Raised	8 (5.13%)	21 (87.5%)	
Mean ± SD median (min, max)	2.09 ± 1.37 1.90 (0.10, 9.18)	6.19 ± 1.72 5.90 (3.20, 11.70)	<0.001^MW^*

**Figure 7:**
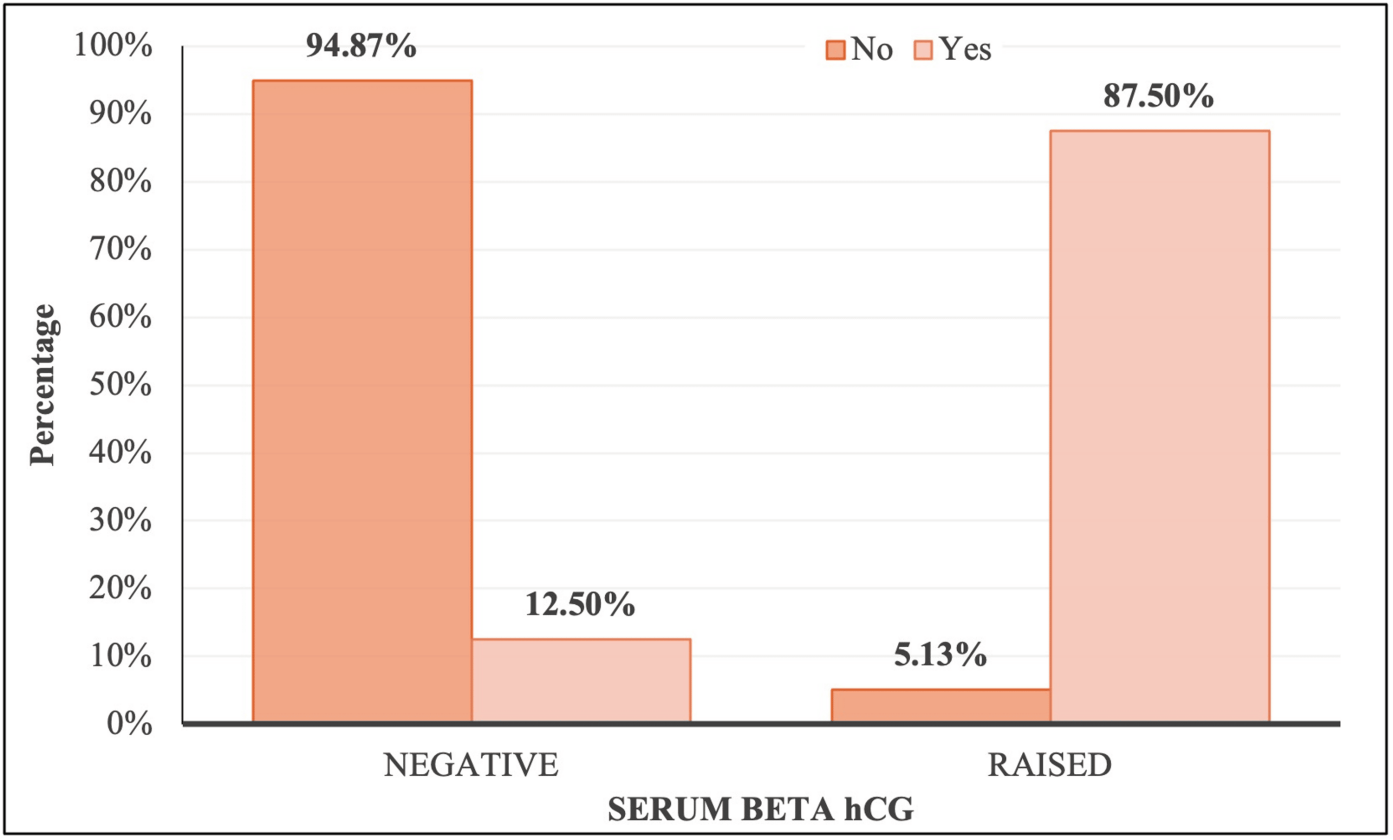
Distribution of serum β-hCG over metastases

**Figure 8:**
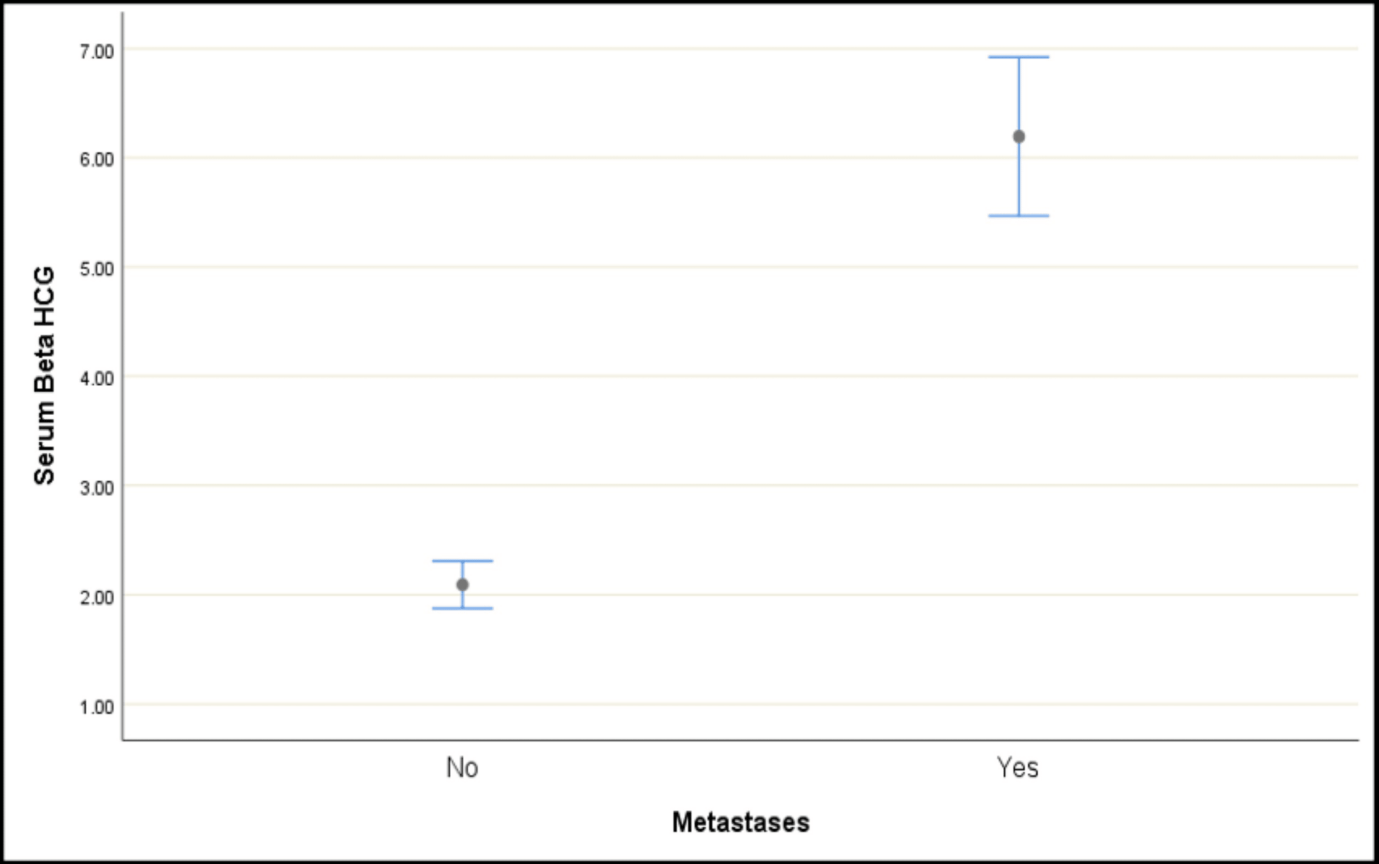
Mean plot of serum β-hCG over metastases β-hCG: beta-human chorionic gonadotropin

Both Fisher’s exact test and the Mann-Whitney U test confirmed a significant difference in serum β-hCG levels based on metastasis status (p-value < 0.001). These findings suggest a strong association between elevated β-hCG levels and the presence of metastases (Figures 7, 8).

Table 4 shows the diagnostic analysis of serum β-hCG for predicting lesion type.

**Table 4:** Diagnostic analysis of serum β-hCG for predicting lesion type β-hCG: beta-human chorionic gonadotropin; PPV: positive predictive value; NPV: negative predictive value; AU-ROC: area under the receiver operating characteristic curve; * indicates statistical significance

	Serum β-hCG
Cutoff	(>) 2.20
Sensitivity (95% CI)	83.67% (74.84%, 90.37%)
Specificity (95% CI)	78.05% (67.54%, 86.44%)
PPV (95% CI)	82% (72.73%, 89.29%)
NPV (95% CI)	80% (69.90%, 87.76%)
AU-ROC (95% CI)	0.830 (0.767, 0.893)
p-value	<0.001*

**Figure 9:**
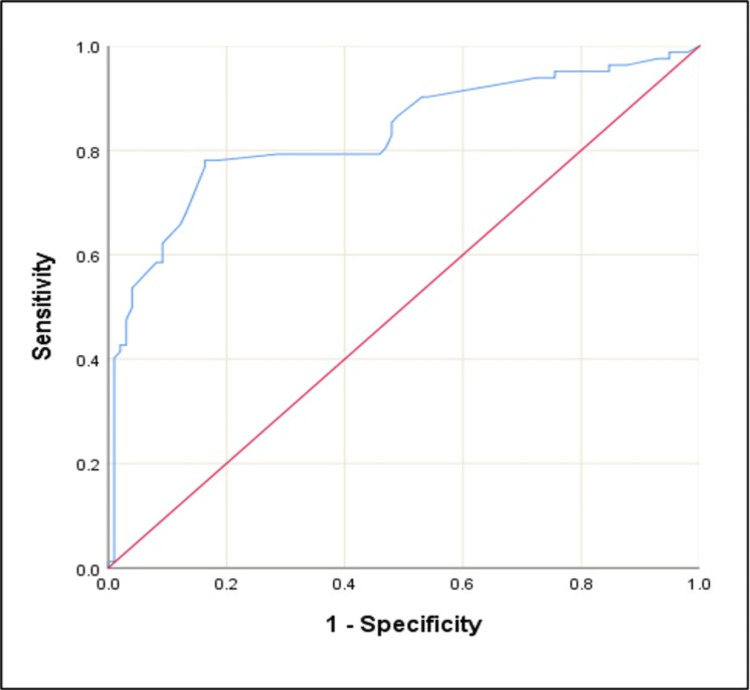
ROC curve of serum β-hCG for predicting lesion type ROC: receiver operating characteristic

The AU-ROC for serum β-hCG is 0.830 at a cutoff of >2.20 with 83.67% sensitivity and 78.05% specificity in predicting lesion type. From logistic regression, we observe that serum β-hCG significantly predicts lesion type (p-value < 0.001) (Figure 9). 

## Discussion

Breast cancer remains the most frequently diagnosed malignancy in India and is a leading cause of mortality among women globally. In recent years, its incidence has shown a marked increase, with a growing number of cases reported in younger women. Numerous factors contribute to the onset and progression of this disease, including genetic predisposition, age, lifestyle changes, tumor size, axillary lymph node involvement, hormone receptor expression, and histological grade. Biomarkers such as β-hCG have emerged as potentially valuable tools in therapeutic decision-making and prognosis prediction in breast cancer management.

hCG, a hormone primarily associated with pregnancy, shows a complex and dual role in breast cancer, with both protective and tumor-promoting properties, depending on its source and context. Sheth et al. found that β-hCG was present in only a small number of breast cancer patients and absent in those with benign breast conditions [14]. Tormey et al. showed that elevated hCG levels alongside other markers helped identify abnormalities in many metastatic and postsurgical patients [15]. Hudelist et al. further noted increased β-hCG/LH receptor expression in invasive tumors, suggesting a role in cancer progression [16]. Mohammed et al. reported that although hCG levels usually remained below standard cutoffs, values above 2 mIU/mL were associated with more advanced disease and poorer prognosis (9). Additionally, Toniolo et al. found that higher early-pregnancy hCG levels correlated with a reduced breast cancer risk later in life, especially for women under 25, with the protective effect influenced by the age at diagnosis and time since pregnancy-offering greater protection when cancer occurred after age 40 or more than 10 years postpartum, but possibly increasing risk for cancers diagnosed soon after pregnancy in younger women [17].

According to Schüler-Toprak et al., naturally occurring placental hCG supports the normal growth and regulation of breast tissue, which may help protect against cancer. In contrast, tumor-derived β-hCG appears to facilitate cancer progression and is associated with unfavorable clinical outcomes [18]. Gehring et al. reinforced this idea through a meta-analysis, suggesting that hCG promotes breast tissue differentiation and may lower cancer vulnerability, though its overall biological function is still not fully understood [19]. Kölbl et al. highlighted hCG’s dual utility as a diagnostic marker and a potential therapeutic target, emphasizing its complex role in tumor growth and metastasis [20]. The findings of this study are in concordance with Janssens et al. [21], who also included a cohort of 25 menopausal women. In our cohort, 1.67% reported a positive family history of breast cancer, whereas Liu et al. [22] observed a 5% prevalence in their study. While Li et al. [23] reported no association between tobacco/alcohol use and breast cancer, our data showed that 6.11% of participants had such habits. Bilateral breast cancer was observed in 9.44% of patients, closely aligning with Donovan’s findings of a 6% bilateral involvement [24]. Multiple breast lesions were found in 7.78% of our cases, whereas Chu et al. reported a 17.6% rate in a larger cohort of 5,758 cases [25].

Among nonmetastatic patients, 94.87% exhibited normal β-hCG levels, while 5.13% had elevated levels. In contrast, 87.5% of those with metastases presented with significantly increased serum β-hCG. The average β-hCG level was 2.09 ± 1.37 in the nonmetastatic group and markedly higher, at 6.19 ± 1.72, in those with metastases. These intergroup differences had a p-value < 0.001 (statistically significant), suggesting a strong correlation between elevated β-hCG levels and metastatic disease. These results align with previous work, including that of Mohammed et al. [9] and Reimer et al. [26]. 

This study, however, has certain limitations that should be pointed out. This study was conducted at a single tertiary care center with a relatively modest sample size (n = 180), which may limit the generalizability of the findings to broader populations. Additionally, serum β-hCG levels can be influenced by various nonmalignant physiological and pathological conditions, some of which may not have been fully excluded, potentially affecting the specificity of the marker. The lack of longitudinal follow-up data restricts the ability to assess β-hCG trends over time and its utility in monitoring disease progression or treatment response. Furthermore, the study did not incorporate a multi-marker panel, thereby limiting comparative evaluation with other established tumor markers such as CA 15-3 or CEA.

## Conclusions

The present study demonstrates that malignant breast lesions have a statistically significantly elevated serum β-hCG compared to benign lesions, with particularly higher levels observed in those with metastatic disease. A β-hCG cutoff value of >2.20 yielded a sensitivity of 83.67% and specificity of 78.05% for predicting malignancy, highlighting its potential as a supportive, noninvasive biomarker. Furthermore, logistic regression analysis reinforced its role as an independent predictor of malignancy. These findings suggest that serum β-hCG may aid in the initial stratification of breast lesions, particularly as preliminary evidence for potential utility where access to advanced imaging or biopsy is delayed. It may also carry prognostic significance in identifying patients at greater risk of metastatic disease, thereby informing treatment planning and follow-up strategies.
